# Supplementary material for: GraphMHC: Neoantigen prediction model applying the graph neural network to molecular structure
Source: PLoS One. 2024 Mar 27;19(3):e0291223. doi: 10.1371/journal.pone.0291223 (PMC10971776; doi:10.1371/journal.pone.0291223)
Supplement: S1 Table — (PDF) [file pone.0291223.s001.pdf]

Supplementary Table S1: SMILES representation of MHC and peptides

	MHC	Peptide
HLA type	HLA-A*01:01	
Amino acid sequence	YFAMYQENMAHTDANTLYIIYRDYTWVARVYRGY	ASFCGSPY
SMILES representation	CC[C@H](C)[C@H](NC(=O)[C@H](Cc1ccc(O)cc1)NC(=O)[C@H](CC(C)C)NC(=O)[C@@H](NC(=O)[C@H](CC(N)=O)NC(=O)[C@H](C)NC(=O)[C@H](CC(=O)O)NC(=O)[C@@H](NC(=O)[C@H](Cc1c[nH]cn1)NC(=O)[C@H](C)NC(=O)[C@H](CCSC)NC(=O)[C@H](CC(N)=O)NC(=O)[C@H](CCC(=O)O)NC(=O)[C@H](CCC(N)=O)NC(=O)[C@H](Cc1ccc(O)cc1)NC(=O)[C@H](CCSC)NC(=O)[C@H](C)NC(=O)[C@H](Cc1ccccc1)NC(=O)[C@@H](N)Cc1ccc(O)cc1)[C@@H](C)O)[C@@H](C)O)C(=O)N[C@H](C(=O)N[C@@H](Cc1ccc(O)cc1)C(=O)N[C@@H](CCCNC(=N)N)C(=O)N[C@@H](CC(=O)O)C(=O)N[C@@H](Cc1ccc(O)cc1)C(=O)N[C@H](C(=O)N[C@@H](Cc1c[nH]c2ccccc12)C(=O)N[C@H](C(=O)N[C@@H](C)C(=O)N[C@@H](CCCNC(=N)N)C(=O)N[C@H](C(=O)N[C@@H](Cc1ccc(O)cc1)C(=O)N[C@@H](CCCNC(=N)N)C(=O)NCC(=O)N[C@@H](Cc1ccc(O)cc1)C(=O)O)C(C)C)C(C)C)[C@@H](C)O)[C@@H](C)CC	C[C@H](N)C(=O)N[C@@H](CO)C(=O)N[C@@H](Cc1ccccc1)C(=O)N[C@@H](CS)C(=O)NCC(=O)N[C@@H](CO)C(=O)N1CCC[C@H]1C(=O)N[C@@H](Cc1ccc(O)cc1)C(=O)O
