# Supplementary material for: GraphMHC: Neoantigen prediction model applying the graph neural network to molecular structure
Source: PLoS One. 2024 Mar 27;19(3):e0291223. doi: 10.1371/journal.pone.0291223 (PMC10971776; doi:10.1371/journal.pone.0291223)
Supplement: S2 Table — Based on the binding affinity provided by the IEDB, non-binding is defined as IC50 ≤500 nM and binding as IC50 >500 nM. Statistics are expressed from the median (the 1st quartile—the 3rd quartile). (PDF) [file pone.0291223.s002.pdf]

Supplementary Table S2: Measurement statistics for all MHC-peptide graphs

| Measure | Non-binding ($n = 116,180$) | Binding ($n = 40,904$) | $p$ of Wilcoxon rank-sum test |
|---|---|---|---|
| Number of nodes | 737(724-750) | 739(727-750) | $< 0.0001$ |
| Number of edges | 1502(1474-1528) | 1506(1482-1530) | $< 0.0001$ |
| Mean degree | 4.08(4.07-4.08) | 4.08(4.07-4.09) | $< 0.0001$ |

Based on the binding affinity provided by the IEDB, non-binding is defined as $IC_{50} \leq 500$ nM and binding as $IC_{50} > 500$ nM. Statistics are expressed from the median (the 1st quartile - the 3rd quartile).
